# Association of area- and volumetric-mammographic density and breast cancer risk in women of Asian descent: a case control study

**DOI:** 10.1186/s13058-024-01829-2

**Published:** 2024-05-15

**Authors:** Shivaani Mariapun, Weang-Kee Ho, Mikael Eriksson, Nur Aishah Mohd Taib, Cheng-Har Yip, Kartini Rahmat, Per Hall, Soo-Hwang Teo

**Affiliations:** 1 Cancer Research Malaysia, Subang Jaya, Selangor Malaysia; 2 School of Mathematical Sciences, Faculty of Science and Engineering, University of Nottingham Malaysia, Semenyih, Selangor Malaysia; 3 Department of Medical Epidemiology and Biostatistics, Karolinska Institutet, Stockholm, Sweden; 4 Faculty of Medicine, University Malaya Cancer Research Institute, University Malaya, Kuala Lumpur, Malaysia; 5 Department of Surgery, Faculty of Medicine, University of Malaya, Kuala Lumpur, Malaysia; 6 Subang Jaya Medical Centre, Subang Jaya, Malaysia; 7 Biomedical Imaging Department, Faculty of Medicine, Universiti Malaya Research Imaging Centre, University of Malaya, Kuala Lumpur, Malaysia; 8 Department of Oncology, Södersjukhuset, Stockholm, Sweden; 9 Department of Public Health and Primary Care, University of Cambridge, Cambridge, UK

**Keywords:** Mammographic density, Asian, Breast cancer

## Abstract

**Background:**

Mammographic density (MD) has been shown to be a strong and independent risk factor for breast cancer in women of European and Asian descent. However, the majority of Asian studies to date have used BI-RADS as the scoring method and none have evaluated area and volumetric densities in the same cohort of women. This study aims to compare the association of MD measured by two automated methods with the risk of breast cancer in Asian women, and to investigate if the association is different for premenopausal and postmenopausal women.

**Methods:**

In this case–control study of 531 cases and 2297 controls, we evaluated the association of area-based MD measures and volumetric-based MD measures with breast cancer risk in Asian women using conditional logistic regression analysis, adjusting for relevant confounders. The corresponding association by menopausal status were assessed using unconditional logistic regression.

**Results:**

We found that both area and volume-based MD measures were associated with breast cancer risk. Strongest associations were observed for percent densities (OR (95% CI) was 2.06 (1.42–2.99) for percent dense area and 2.21 (1.44–3.39) for percent dense volume, comparing women in highest density quartile with those in the lowest quartile). The corresponding associations were significant in postmenopausal but not premenopausal women (premenopausal versus postmenopausal were 1.59 (0.95–2.67) and 1.89 (1.22–2.96) for percent dense area and 1.24 (0.70–2.22) and 1.96 (1.19–3.27) for percent dense volume). However, the odds ratios were not statistically different by menopausal status [*p* difference = 0.782 for percent dense area and 0.486 for percent dense volume].

**Conclusions:**

This study confirms the associations of mammographic density measured by both area and volumetric methods and breast cancer risk in Asian women. Stronger associations were observed for percent dense area and percent dense volume, and strongest effects were seen in postmenopausal individuals.

**Supplementary Information:**

The online version contains supplementary material available at 10.1186/s13058-024-01829-2.

## Background

Mammographic density (MD) reflects the composition of fibro-glandular tissue of the breast, as visualised on a mammogram. MD is an independent predictor of breast cancer risk, although the strength of its association varies across studies, due in part to the different methods of MD assessment and different partitioning thresholds used to define high and low MD [1–3]. Efforts to make measuring MD less reader-dependent and more reproducible have resulted in the development of a number of fully-automated methods for measuring MD [4–6], including both volumetric and area-based assessments methods.

In women of European ancestry Volumetric assessments of density have been shown to be a stronger predictor of risk compared to area-based density [7, 8]. Volumetric methods are less influenced by compression force and are more sensitive to breast thickness, and may more accurately estimate the amount of fibroglandular tissue for women with larger breasts [9–11]. However Asian women have smaller and denser breasts compared to women of European ancestry, and the performance of area and volume-based densities have hitherto not been compared in the same study.

In this study, we aim to determine and compare the effects of two automated MD measures, namely STRATUS measurements of area densities, and Volpara measurements of volumetric densities, on breast cancer risk in the Asian population, and to explore the potential variation by menopausal status.

## Methods

### Study participants, data collection and eligibility criteria

Cases comprised of patients who were recruited sequentially into the Malaysian Breast Cancer Genetics (MyBrCa) study from Subang Jaya Medical Centre (SJMC), between 2012 and 2020, and University Malaya Medical Centre (UMMC), between 2003 and 2020. Controls were women between 40 and 74 years old with no prior history of breast cancer that were recruited into the Malaysian Mammography Study (MyMammo) from the same participating hospitals as cases. The study details have been previously published [12]. All participants answered a detailed questionnaire which included information on lifestyle and reproductive risk factors, socio-demographic factors, and family history and provided blood sample for genetic testing.

Bilateral full-field digital mammograms (FFDMs) for cases were retrieved from the medical image storage servers retrospectively starting in June 2018 and for controls were collected at recruitment. The bilateral cranio-caudal (CC) and medio-lateral oblique (MLO) views for both raw and processed images, where possible, were retrieved. Cases were excluded from the research study if: (a) digital mammograms were conducted more than 12 months prior to cancer diagnosis, (b) only mammograms ipsilateral to the breast cancer were available. Controls were excluded from the research study if no mammograms were available for analysis. All participants included in the study were of self-declared Chinese, Malay or Indian ethnicity and had information on age at mammography, body mass index (BMI) and/or menopausal status. In total, 10% of cases and 69% of controls were available and eligible for matching.

### Matching

For the case–control analysis of mammographic density and breast cancer risk, as raw and processed images were not available for all women, cases and controls in the full dataset were matched for age (within 5 years) and ethnicity (exact) separately for the analyses of STRATUS, which measures processed images, and Volpara, which measures raw images. Age-matching was performed in each ethnic group using a 1:4 case to control ratio nearest neighbour propensity score matching using the *matchit* package in *R*. For the STRATUS study, a total of 488 cases and 1796 controls were included in the matched case–control study, of which 82.2% of cases were matched to four controls, 9% to three controls, 3.5% to two controls and 5.3% to only one control. For the Volpara study, a total of 436 cases and 1623 controls were included, of which 81.4% were matched to four controls, 12.4% to three controls, 3.2% to two controls and 3% to only one control. In total, 531 cases and 2297 controls were included for analysis of which data was available for both STRATUS and Volpara in 393 cases and 1122 controls.

### Mammographic density (MD) assessments

Mammography was performed using machines from three different manufacturers; Hologic [Models: Lorad Selenia, Selenia Dimensions and Tomo Selenia Dimensions], General Electric (GE) Senographe Essential, and Siemens Mammomat Novation. Area-based MD was determined using STRATUS, a fully automated machine-learning method for assessing MD based on image features assessed using thresholding methods, by the developers of STRATUS at the Karolinska Institute, Sweden [4]. Volumetric MD was computed using Volpara Data Manager version 1.1.109 [5]. Six MD phenotypes were considered in this study: absolute dense area (DA) and volume (DV), percent dense area (PDA, i.e., absolute dense area/total breast area) and volume (PDV, i.e., absolute dense volume/total breast volume), and non-dense area (NDA) and volume (NDV). We also categorised MD according to the computer-generated BI-RADS scores (cBIRADS) generated by STRATUS, and the clinical classification score (Volpara Density Grades (VDG)).

#### Image laterality

Pearson’s correlation coefficients and previous studies showed that there were strong correlations between CC and MLO measurements [13, 14]. The Wilcoxon rank sum test was performed to compare the distribution of MD in the left and right mammograms in the control group. For the CC view mammograms, percent dense volume was higher in the right breast (Left median 9.1%; Right 9.5%, *P* = 0.035), whereas for the MLO view, three measures were higher in the left breast [dense volume (Left 57.6 cm^3^; Right 56.5 cm^3^, *P* = 0.006), non-dense volume (Left 591.8 cm^3^; Right 561.7 cm^3^, *P* = 0.011) and total breast volume (Left 653.4 cm^3^; Right 628.0 cm^3^, *P* = 0.006)]. As there was less variation in MD measurements for the CC view, MD measurements from the CC view mammograms of unaffected breasts of cases were used in all analyses, and matched by laterality in the controls.

### Statistical analyses

Box-Cox transformation was used to transform MD phenotypes into approximately normal distribution.

#### Confounder selection

Covariates that were assessed include socio-demographic factors, known lifestyle and reproductive risk factors of breast cancer, mammogram machine and compressed breast thickness. A covariate was considered confounding if: (a) it was significantly associated with MD in controls at *P* < 0.05, after accounting for other associated variables; (b) it was significantly associated with breast cancer risk at *P* < 0.05, after accounting for other associated variables; and (c) it had a magnitude of confounding that was greater than 5%.

Age at first full term pregnancy, total number of live births and breast feeding were only evaluated among parous women. Parous women were defined as those who have had at least one full-term pregnancy. The use of hormone replacement therapy (HRT) was only evaluated among postmenopausal women. Postmenopausal women were defined as women who have not had their periods for at least 12 months prior to their enrolment into the study or if they self-reported that they were postmenopausal at enrolment.

#### Association of mammographic density (MD) phenotypes and breast cancer risk

We assessed the association between mammographic density phenotypes (treated either as continuous or categorical variables) and breast cancer risk using conditional logistic regression, adjusting for selected confounders. When MD was treated as a continuous variable, odds ratios per-adjusted standard deviations (OPERA [15]) was calculated to allow comparison across MD phenotypes. When MD was treated as a categorical variable, MD phenotypes were categorised into four equal quartiles based on the MD distribution in controls, using the first quartile as the reference group. We also categorised MD according to the computer-generated BI-RADS scores, cBIRADS, generated by STRATUS, and Volpara Density Grades (VDG), which is the classification used to report density, measured by Volpara, in the clinic. Weighted kappa, using quadratic weighting, was calculated to assess the concordance between quantiles of STRATUS and Volpara measurements.

The association between MD phenotypes and breast cancer risk by menopausal status were conducted using unconditional regression. Z-tests were conducted to determine whether the odds ratios for mammographic densities and breast cancer risk were different for premenopausal and postmenopausal women.

All statistical analyses were performed with R version 3.6.1.

## Results

### Characteristics of study participants

**Fig. 1:**
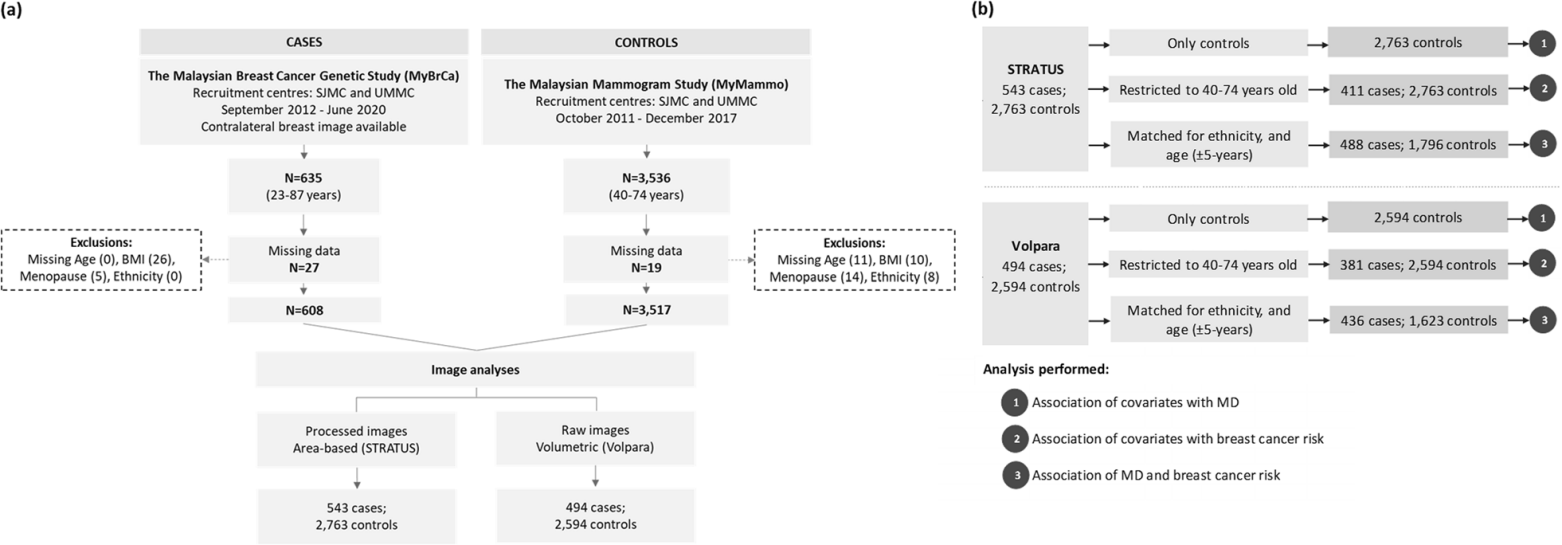
Flowchart illustrating **a** the selection of cases and controls for mammographic density (MD) assessment by STRATUS and Volpara, and **b** participants included in the different analyses performed including the analysis of (1) the association of covariates with MD, (2) the association of covariates with breast cancer risk, and (3) the association of MD and breast cancer risk

Participant selection and descriptive statistics of cases and controls are presented in Fig. 1 and Table 1. The majority of controls within the STRATUS study (67.7%) were recruited from the private tertiary hospital (SJMC), while approximately half of the controls within the Volpara study were from the government-funded teaching hospital (UMMC). Most of the mammograms were obtained from the Hologic machine.

**Table 1:** Characteristics of controls and cases with STRATUS and Volpara measurements available (unmatched dataset)

Characteristic	Controls						
		STRATUS			Volpara		
	STRATUS, N; Volpara, N	Mean/N	SD/%	Range	Mean/N	SD/%	Range
Age at interview/diagnosis, years	2763; 2594	53.0	8.0	40.0–74.0	54.0	8.4	40.0–74.0
Age at mammogram, years	2763; 2594	52.7	8.1	40.0–74.0	54.0	8.4	40.0–74.0
BMI, kg/m^2^	2763; 2594	25.2	4.6	15.6–51.1	25.6	4.8	15.0–51.1
Ethnicity	2763; 2594						
Chinese		1681	60.8		1374	53.0	
Malay		510	18.5		643	24.8	
Indian		572	20.7		577	22.2	
Centre	2763; 2594						
SJMC		1870	67.7		1122	40.6	
UMMC		893	32.3		1472	53.3	
Education	2648; 2415						
Primary or less		227	8.6		227	9.4	
Secondary		1379	52.1		1280	53.0	
Tertiary		1042	39.4		908	37.6	
*Missing*^a^		115	4.2		179	6.5	
Income, RM	2702; 2522						
< 5000		1486	55.0		1506	59.7	
5–10,000		759	28.1		661	26.2	
> 10,000		457	16.9		355	14.1	
*Missing*^a^		61	2.2		72	280	
Age of menarche, years	2742; 2581	12.9	1.4	8.0–18.0	12.9	1.4	8.0–18.0
Menopausal status	2763; 2594						
Premenopausal		1178	42.6		982	37.9	
Postmenopausal		1585	57.4		1612	62.1	
Parity	2763; 2594						
Nulliparous		459	19.9		460	17.7	
Parous		2304	83.4		2134	83.3	
* Missing*^a^		0	0.0		0	0.0	
Number of live births	2763; 2594	2.3	1.5	0–8.0	2.3	1.5	0–8.0
Age at first live birth^b^, years	2239; 2047	27.4	4.7	14.0–52.0	27.1	4.7	14.0^9.0
Breastfeeding^b^	2182; 2065						
Never		475	21.8		423	20.5	
< 12 months		1178	54.0		1071	51.9	
> 12 months		529	24.2		571	27.7	
*Missing*^a^		122	5.3		69	3.2	
HRT use^c^	1577; 1605						
Never		1234	78.2		1230	76.6	
Ever		343	21.8		375	23.4	
Past		288	18.3		310	19.3	
Current		55	3.5		65	4.0	
*Missing*^a^		8	0.5		7	4.3	
Oral contraceptives	2750; 2587						
Never		1944	70.7		1830	70.7	
Ever		806	29.3		757	29.3	
Past		772	28.1		736	28.4	
Current		34	1.2		21	0.8	
* Missing*^a^		13	0.5		7	0.3	
Regular alcohol intake^d^	2688; 2591						
No		2531	94.2		2261	87.3	
Yes		157	5.8		330	12.7	
*Missing*^a^		75	2.7		3	0.1	
Smoking	2758; 2593						
Never		2577	93.4		2483	95.8	
Ever		181	6.6		110	4.2	
Past		134	4.9		81	3.2	
Current		47	1.7		29	1.1	
*Missing*^a^		5	0.2		1	0.04	
Family history of breast cancer	2761; 2594						
None reported		1871	67.8		1689	65.1	
1st degree		344	12.5		357	13.8	
1st/2nd degree		546	19.8		548	21.1	
*Missing*^a^		2	0.1		0	0	
Mammogram system	2763; 2594						
Hologic		2645	95.7		2213	85.3	
GE		118	4.3		229	8.8	
Siemens		0	0		152	5.9	
Hormone receptor status	na						
ER + ve		–	–		–	–	
ER −ve		–	–		–	–	
*Missing*^a^		–	–		–	–	
Cancer laterality	na						
Left		–	–		–	–	
Right		–	–		–	–	
Mammographic density^e^				Median (IQR)			Median (IQR)
Dense area, cm^2^	2763; na	29.4	23.8	24.3 (12.4–40.5)			
Percent dense area, cm^2^	2763; na	27.5	18.7	26.1 (11.7–41.2)			
Non-dense area, cm^2^	2763; na	89.4	54.8	75.1 (49.6–115.6)			
Total breast area, cm^2^	2763; na	118.9	53.1	108.8 (80.4–144.9)			
Dense volume, cm^3^	na; 2594				53.5	30.8	46.7(32.7–66.3)
Percent dense volume, cm^3^	na; 2594				11.7	7.4	9.3(6.2–15.6)
Non-dense volume, cm^3^	na; 2594				508.2	318	442.6(274.1–654.0)
Total breast volume, cm^3^	na; 2594				561.7	326	493.0(328.7–719.5)

Characteristic	Cases							Case vs. Controls^f^	
		STRATUS			Volpara				
	STRATUS, N; Volpara, N	Mean/N	SD/%	Range	Mean/N	SD/%	Range	*p* value STRATUS	*p* value Volpara
Age at interview/diagnosis, years	543; 494	51.2	12.2	22.0–87.0	51.6	12.2	22.0–83.0	**0.001**	** < 0.001**
Age at mammogram, years	543; 494	51.0	12.2	22.0–87.0	51.6	12.2	22.0–83.0	**0.002**	** < 0.001**
BMI, kg/m^2^	543; 494	23.3	4.1	15.4–42.2	23.5	4.1	15.4–42.0	** < 0.001**	** < 0.001**
Ethnicity	543; 494							** < 0.001**	** < 0.001**
Chinese		470	86.6		407	82.0			
Malay		43	7.9		52	10.5			
Indian		30	5.5		35	7.1			
Centre	543; 494							** < 0.001**	** < 0.001**
SJMC		535	98.5		463	93.7			
UMMC		8	1.5		31	6.3			
Education	466; 394							** < 0.001**	** < 0.001**
Primary or less		66	14.2		53	13.5			
Secondary		181	38.8		145	36.8			
Tertiary		219	47.0		196	49.7			
*Missing*^a^		77	14.2		100	20.2			
Income, RM	465; 390							**0.006**	** < 0.001**
< 5000		238	51.2		190	48.7			
5–10,000		120	25.8		103	26.4			
> 10,000		107	23.0		97	24.9			
* Missing*^a^		78	14.4		104	26.4			
Age of menarche, years	473; 424	12.8	1.4	8.0–18.0	12.7	1.4	8.0–18.0	0.065	**0.016**
Menopausal status	543; 494							**0.001**	** < 0.001**
Premenopausal		272	50.1		239	48.4			
Postmenopausal		271	49.9		255	51.6			
Parity	543; 493							**0.001**	**0.022**
Nulliparous		123	22.7		109	22.1			
Parous		420	77.3		384	77.9			
* Missing*^a^		0	0		1	0.2			
Number of live births	543; 492	2.1	1.7	0.0–11.0	2.1	1.6	0.0–8.0	**0.026**	**0.025**
Age at first live birth^b^, years	419; 383	27.6	5.0	14.0–47.0	27.5	4.90	14.0–44.0	** < 0.001**	** < 0.001**
Breastfeeding^b^	339; 287							** < 0.001**	** < 0.001**
Never		97	28.6		75	25.9			
< 12 months		213	62.8		187	65.5			
> 12 months		29	8.6		25	8.6			
*Missing*^a^		81	19.3		97	27.6			
HRT use^c^	227; 210							** < 0.001**	** < 0.001**
Never		200	88.1		187	89.0			
Ever		27	11.9		23	11.0			
Past		27	11.9		23	11.0			
Current		0	0.0		0	0.0			
*Missing*^a^		44	19.4		45	19.8			
Oral contraceptives	484; 421							0.126	0.106
Never		369	76.2		314	74.60			
Ever		115	23.8		107	25.40			
Past		113	23.3		107	25.40			
Current		2	0.4		0	0.00			
* Missing*^a^		59	10.9		73	14.80			
Regular alcohol intake^d^	387; 401							0.179	** < 0.001**
No		435	92.6		322	80.3			
Yes		35	7.4		79	19.7			
*Missing*^a^		73	13.4		93	18.8			
Smoking	496; 416							0.369	** < 0.001**
Never		458	92.3		380	91.3			
Ever		38	7.7		36	8.7			
Past		30	6.0		28	6.7			
Current		8	1.6		8	1.9			
*Missing*^a^		47	8.7		78	15.8			
Family history of breast cancer	539; 395							**0.007**	**0.005**
None reported		435	80.7		313	79.2			
1st degree		45	8.3		38	9.6			
1st/2nd degree		104	19.3		82	20.8			
*Missing*^a^		4	0.7		99	20.0			
Mammogram system	543; 494							** < 0.001**	** < 0.001**
Hologic		493	90.8		494	100.0			
GE		0	0		0	0			
Siemens		50	9.2		0	0			
Hormone receptor status	526; 434								
ER + ve		354	67.3		292	67.3			
ER −ve		172	32.7		142	32.7			
*Missing*^a^		17	3.1		60	12.1			
Cancer laterality	543; 494								
Left		274	50.5		249	50.4			
Right		269	49.5		245	49.6			
Mammographic density^e^				Median (IQR)			Median (IQR)		
Dense area, cm^2^	543; na	32.6	25.6	27.0 (14.8–43.9)				**0.006**	
Percent dense area, cm^2^	543; na	33.3	20.0	33.8 (17.2–47.6)				** < 0.001**	
Non-dense area, cm^2^	543; na	70	43.8	57.7 (38.9–84.5)				** < 0.001**	
Total breast area, cm^2^	543; na	102.6	45.0	94.0 (70.4–124.1)				** < 0.001**	
Dense volume, cm^3^	na; 494				59.4	34.7	50.9 (34.0–75.4)		** < 0.001**
Percent dense volume, cm^3^	na; 494				15.1	8.5	12.9 (8.2–20.6)		** < 0.001**
Non-dense volume, cm^3^	na; 494				404.8	276.3	340.9 (207.7–524.1)		** < 0.001**
Total breast volume, cm^3^	na; 494				464.2	289.3	403.6 (272.6–570.1)		** < 0.001**

Means and standard deviations are provided for continuous variables and frequencies and percentages are provided for categorical variables. *P* < 0.05 are written in bold font

N, numbers or frequencies; SD, standard deviation; BMI, body mass index; SJMC, subang jaya medical centre; UMMC, university malaya medical centre; HRT; hormone replacement therapy; ER, estrogen receptor; IQR, interquartile range

^a^The percentage of missingness is calculated by dividing the number of participants with missing data for the characteristic of interest with the total number of samples available for analysis i.e., N = 2763 and N = 2594 for the STRATUS and Volpara studies respectively.

^b^Among parous women only

^c^Among postmenopausal women only.

^d^One glass or more per week

^e^Mammographic density measurements from the left and right CC view mammograms were used for controls, and from the unaffected breast mammograms were used for cases

^f^For comparison of cases and controls, tests used were Chi-square (without Yate's correction), Welch 2-sided t-test and Fischer's exact test when n < 5 for at least one cell

### Confounders

We identified potential confounders as covariates with *P* value < 0.05 with both MD phenotypes and breast cancer risk in the multivariable models, and these were breastfeeding for absolute dense area and dense volume, alcohol intake for non-dense volume and breast thickness for all MD phenotypes except dense area (Additional file 1: Table S1). Additionally, although not significant in our study, menopausal status and parity were included as potential confounders as these variables have consistently been reported to be associated with both MD and breast cancer risk in the literature. Of the list of potential confounders, only those resulting in > 5% change in the magnitude of MD association with risk were retained in the model for adjustment. The final list of variables included in the association analyses for adjustment can be found Additional file 1: Table S2.

### Association of mammographic density (MD) phenotypes and breast cancer risk

#### All women

**Fig. 2:**
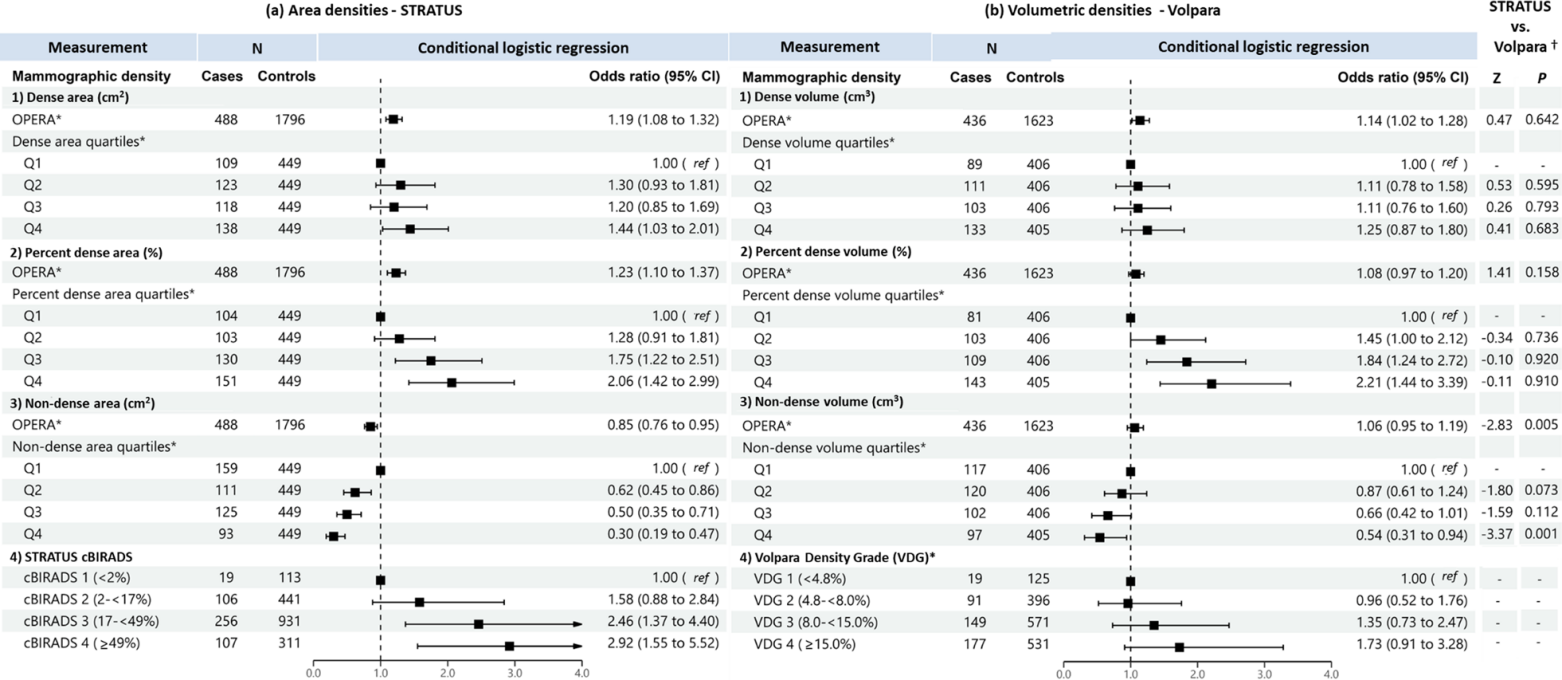
Associations of **a** STRATUS area mammographic densities and **b** Volpara volumetric mammographic densities, with breast cancer risk. *Adjusted for relevant confounding factors. ^†^Z-tests comparing estimated regression coefficients between the STRATUS and Volpara studies

**Fig. 3:**
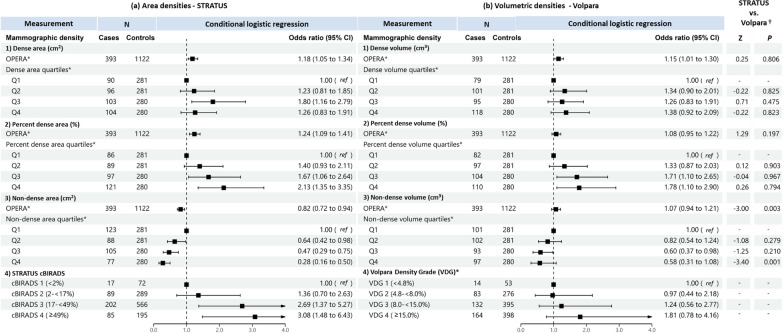
Associations of **a** STRATUS area mammographic densities, **b** Volpara volumetric mammographic densities, with breast cancer risk in the dataset of 393 cases and 1122 controls included in both STRATUS and Volpara studies. *Adjusted for relevant confounding factors. ^†^Z-tests comparing estimated regression coefficients between the STRATUS and Volpara studies

When treated as a continuous variable, both dense area and dense volume were significantly associated with breast cancer risk, the odds per adjusted standard deviation (OPERA) and the corresponding 95% CI were 1.19 (1.08–1.32) and 1.14 (1.02–1.28), respectively (Fig. 2). However, when categorised into quartiles, only the highest quartile of dense area was significantly associated with risk (odds ratio (95% CI) was 1.44 (1.03–1.21)). This association was no longer significant in analyses limited to overlapping samples between the STRATUS and Volpara studies (1.26, 95% CI: 0.83-1.91) (Fig. 3).

For percent density, OPERA for percent dense area was significant (1.23, 95% CI 1.10–1.37) while the OPERA for percent dense volume was not significant (1.08, 95% CI 0.97–1.20). However, quartiles analyses of percent density showed significant association for both MD measurement methods, with risk estimates increased consistently across quartiles. The OR of highest versus lowest quartile was 2.06 (95% CI 1.42–2.99) for percent dense area and 2.21 (95% CI 1.44–3.39) for percent dense volume (Fig. 2). There was no significant difference between the ORs of percent dense area and percent dense volume (p-value of Z-test < 0.05). Similar results were observed for analyses limited to overlapping samples between the STRATUS and Volpara studies (Fig. 3).

Non-dense area was significantly associated with a lower breast cancer risk (OPERA 0.85, 95% CI 0.76–0.95). Risk estimates decreased consistently, from OR 0.62 (95% CI: 0.45-0.86) to 0.50 (95% CI: 0.35-0.71) and 0.30 (95% CI: 0.19-0.47), comparing the first quartile of non-dense area with the second, third and fourth quartile, respectively. By contrast, the OPERA estimate for non-dense volume was not significant (1.06, 95% CI: 0.95-1.19), although the pattern of association for quartiles was similar to that observed for non-dense area i.e., OR 0.87 (95% CI: 0.61-1.24), OR 0.66 (95% CI: 0.42-1.01) and OR 0.54 (95% CI: 0.31-0.94) for the second, third and fourth quartiles respectively, the corresponding strengths of association were weaker (Figs. 2 and 3).

Where densities were categorised according to area-based cBIRADS and volume-based VDG, women in cBIRADS 3 and cBIRADS 4 were associated with a 2.5-fold (*P* = 0.003) and 2.9-fold (*P* < 0.001) greater odds of disease, respectively (Fig. 2a). By contrast, VDG was not associated with breast cancer risk (Fig. 2b). The same pattern was observed for analyses limited to overlapping samples between the STRATUS and Volpara studies (Fig. 3).

There are no appreciable differences between the results generated using the CC and MLO view measurements (Additional file 1: Table S2).

**Fig. 4:**
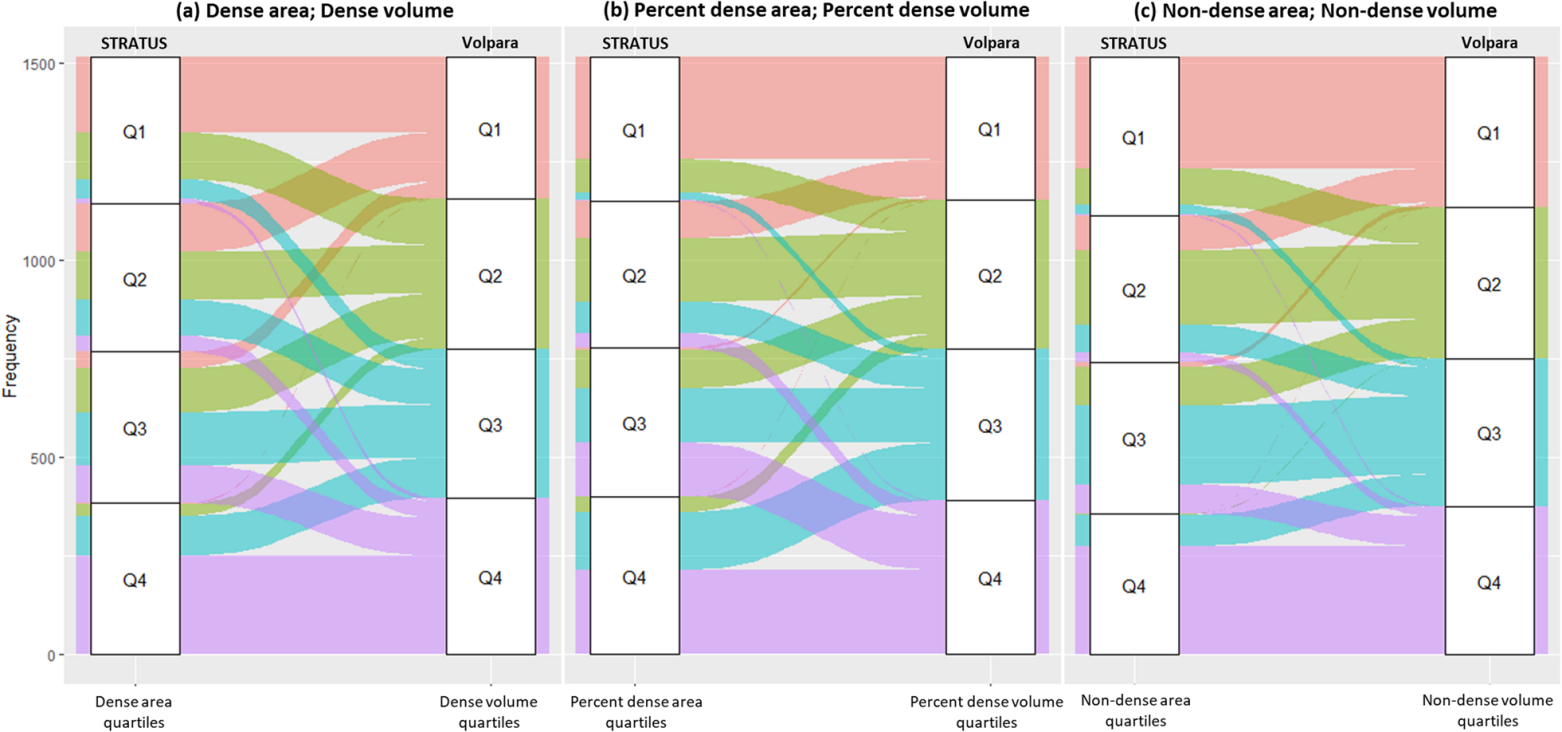
Concordance between the classification of **a** absolute dense area/volume, **b** percent dense area/volume and **c** non-dense area/volume into quartiles using STRATUS and Volpara measurements. *Note:* Agreement between the STRATUS and Volpara measurements for classifying women into mammographic density quartiles was calculated using Cohen’s weighted kappa. Weighted Kappa, κw values for dense area/volume, percent dense area/volume, and non-dense area/volume were 0.28, 0.35 and 0.50, respectively

The agreement between the STRATUS and Volpara measurements for classifying women into mammographic density quartiles was fair for absolute density (Weighted Kappa, κw = 0.28) and percent density (0.35), and moderate for non-dense area and volume (0.50). Figure 4 illustrates the magnitude of concordance for the classification of area and volumetric MD quartiles. Although there is some agreement between STRATUS and Volpara, there are instances of discordance where individuals shift to adjacent quartiles or even skip one quartile altogether.

#### Analyses by menopausal status

**Fig. 5:**
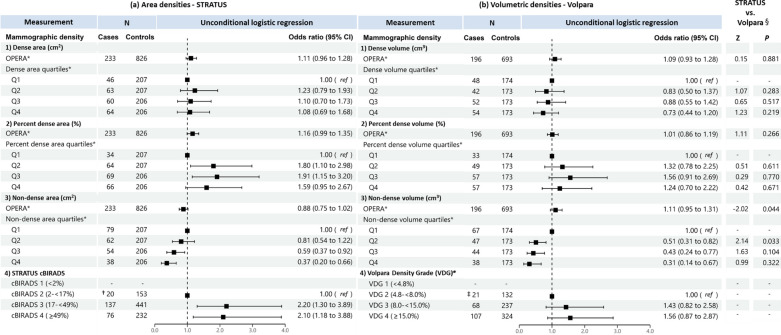
Associations of **a** STRATUS area mammographic densities and **b** Volpara volumetric mammographic densities, with breast cancer risk in premenopausal women. *Adjusted for relevant confounding factors. ^†^Reference category is cBIRADS 1 (< 2%) + cBIRADS 2 (2− < 17%). ‡ Reference category is VDG 1 (< 4.8%) + VDG 2 (4.8—< 8.0%). ^§^Z-tests comparing estimated regression coefficients between the STRATUS and Volpara studies

**Fig. 6:**
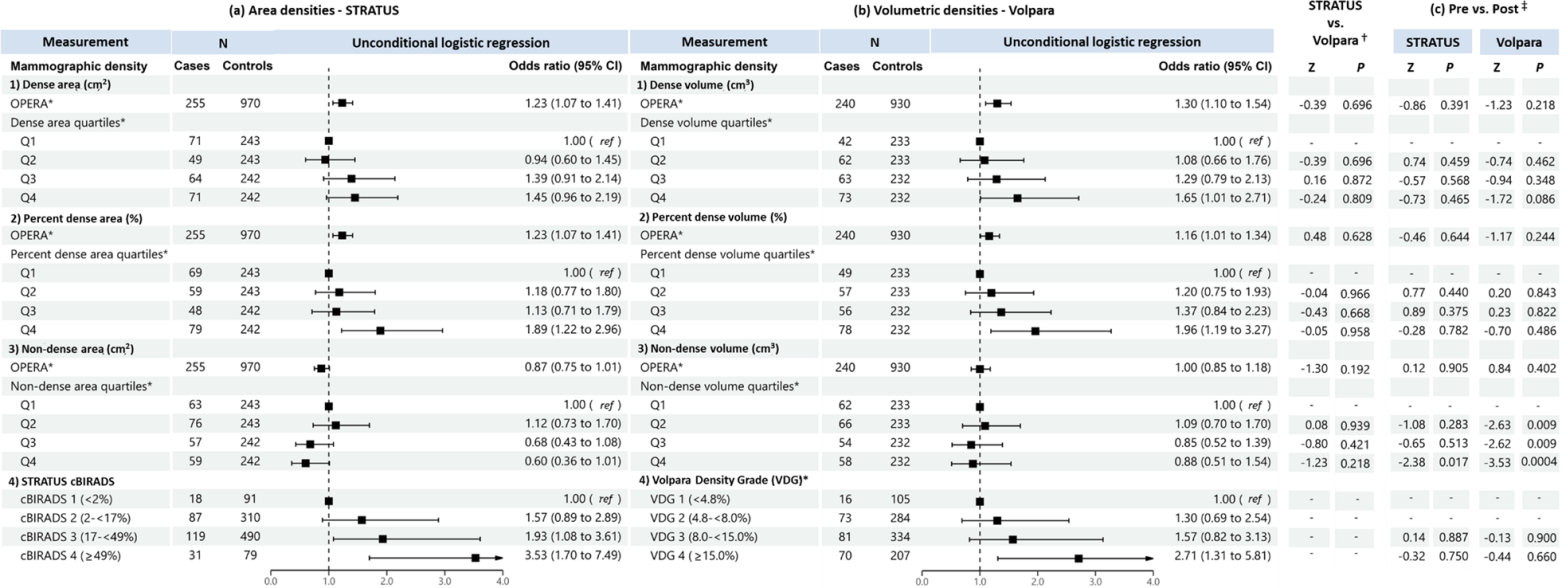
Associations of **a** STRATUS area mammographic densities, **b** Volpara volumetric mammographic densities, with breast cancer risk in postmenopausal women and, **c** comparison of regression coefficients for premenopausal and postmenopausal women. *Adjusted for relevant confounding factors. ^†^Z-tests comparing estimated regression coefficients between the STRATUS and Volpara studies. ^‡^Z-tests comparing estimated regression coefficients between premenopausal and postmenopausal women

Figures 5 and 6 show the association of MD with breast cancer risk for premenopausal and postmenopausal women, respectively. For dense area and dense volume, both OPERAs and quantile analyses were not significantly associated with breast cancer risk in premenopausal women. By contrast, consistent with the all-women analysis, OPERA for both dense area (OR 1.23, 95% CI: 1.07–1.41) and dense volume (OR 1.30, 95% CI: 1.10–1.54) were significant in postmenopausal women, but the corresponding quartile analyses did not show significant associations for dense area and was only significant for the association of the highest dense volume quartile and risk when compared to the lowest dense volume quartile among postmenopausal women (OR 1.65, 95% CI: 1.01-2.71).

For percent density, OPERAs for percent dense area and percent dense volume were significant in postmenopausal women but not premenopausal women. The OPERAs were 1.23 (95% CI 1.07–1.41) and 1.16 (95% CI 1.01–1.34) for precent dense area and percent dense volume, respectively, in postmenopausal women. The corresponding estimates in premenopausal women were 1.16 (95% CI 0.99–1.35) and 1.01 (95% CI 0.86–1.19). The observed significant association of highest versus lowest quartile in all women analysis was replicated in postmenopausal women (OR 1.89, 95% CI: 1.22-2.96 for percent dense area; OR 1.96, 95% CI: 1.19-3.27 for percent dense volume), but not premenopausal women (OR 1.59, 95% CI: 0.95-2.67 for percent dense area; OR 1.24, 95% CI: 0.70-2.22 for percent dense volume).

For non-dense MD phenotype in both premenopausal and postmenopausal women, OPERAs were not significant in both non-dense area (0.88, 95% CI: 0.75-1.02 and 0.87, 95% CI: 0.75-1.01 for premenopausal and postmenopausal women, respectively) and non-dense volume (1.11, 95% CI: 0.95-1.31 and 1.00, 95% CI: 0.85-1.18, respectively) measurements. However, the quartile analyses for both non-dense area and non-dense volume were significant in premenopausal women, comparing the highest and lowest non-dense area quartiles (OR 0.37, 95% CI: 0.20-0.66) and non-dense volume quartiles (OR 0.31, 95% CI: 0.14-0.67), but not postmenopausal women (OR 0.60, 95% CI: 0.36-1.01 for non-dense area; OR 0.88, 95% CI: 0.51-1.54 for non-dense volume).

## Discussion

In this study of women of Asian-ancestry, we found that percent mammographic density is a strong breast cancer risk factor, with similar magnitudes of association for both area and volumetric mammographic density measures. Comparing women in the lowest quartiles, women with percent density in the highest quartiles had approximately two-fold higher odds of breast cancer. The observed association was however significant only in postmenopausal women but not in premenopausal women.

The two-fold risk estimates reported in this study are consistent with those found in a meta-analysis of Japanese, Korean and Singaporean women comprising of one cohort study and five case–control studies, which reported a summary effect size of 2.2 (95% CI 1.5–3.2) [16], as well as with a large meta-analysis of European women using the BI-RADS density four-category classification [3]. The corresponding odds ratio per adjusted standard deviation (OPERA) was similar to a Korean study of 213 cases and 630 controls [17], but lower than those previously reported in women of European ancestry. A study of Australian women reported OPERA of 1.52 (95% CI 1.34–1.73) for percent dense area, compared to 1.23 (95% CI 1.10–1.37) this study, suggesting potential ethnic differences in MD-risk associations [18].

Our findings of lack of MD-risk association in premenopausal women align with similar-sized studies in other Asian populations [19–22]. For instance, a multicentre Japanese study (530 cases, 1043 controls) found a near three-fold increase in breast cancer odds (OR 2.9, 95% CI 1.1–7.2) among postmenopausal women with extremely dense breast (>75% glandular tissue), while no significant association was observed in premenopausal women [19]. Similarly, another study in Japanese women (146 cases, 659 controls) revealed a four-fold higher odds of breast cancer among postmenopausal women with > 75% percent densities, with no significant association in premenopausal women [21]. However, it is important to note that a recent large prospective Korean study comprising of ~ 65,000 breast cancer cases reported that breast density is associated with breast cancer risk in both premenopausal (OR 2.4, 95% CI 2.2–2.5) and postmenopausal (OR 2.9, 95% CI 2.8–3.0) women, suggesting that larger sample sizes in premenopausal women are required to detect a significant association with breast cancer risk [20].

Our study did not yield conclusive evidence regarding the association of absolute MD measures with breast cancer risk. While the odds ratios for continuous dense area and dense volume were significant at a nominal level (1.19, 95% CI 1.08–1.32 and 1.14, 95% CI 1.02–1.28, respectively), the results from quartile analysis did not support the significant associations. We also observed a stronger inverse association with non-dense area compared to non-dense volume that was significant in our analyses of all women and premenopausal women, but not that of postmenopausal women. This inverse association is consistent with previous studies in women of European ancestry reporting a protective effect of having greater amounts of fat or non-dense tissue in the breast [23].

In summary, our study confirms the significance of MD as a robust breast cancer risk factor in Asian-ancestry women, with percent density showing consistent associations across area and volumetric-based measures. However, the lack of MD-risk association in premenopausal women underscores the need for further investigation in larger datasets. While our findings contribute to the understanding of MD and breast cancer risk, the inconclusive evidence regarding absolute MD measures prompts a critical evaluation of their utility in risk prediction models for this population.

This study had several limitations. First, more than 90% of the cases were recruited from one recruitment centre, making it impossible to match cases and controls based on centre. However, we adjusted our analyses for this factor. Second, some covariates have missingness rates greater than 10%, which may explain some of the unexpected results (e.g. the protective effect observed for HRT among postmenopausal women and alcohol consumption). Third, the healthy controls were women attending an opportunistic screening mammography programme and may be enriched for a family history of breast cancer. This is likely to be the reason family history of breast cancer is not associated with breast cancer risk in this study. Finally, only the mammograms performed at the time of cancer detection (or close to cancer detection) were available for the cases. Given that densities measured from the unaffected contralateral breasts have been shown to be similarly associated with risk of disease [8], densities of the contralateral breasts were used as surrogate measurements.

## Conclusions

In conclusion, our study underscores the significance of mammographic density (MD) as a strong predictor of breast cancer risk in women of Asian-ancestry, particularly in postmenopausal individuals. While percent density, for both area- and volume-based measures, consistently demonstrated significant association, absolute MD measures yielded inconclusive results. Future research should aim to elucidate ethnic-specific MD-risk associations and refine risk prediction models to incorporate the most predictive MD measures, thus enabling more targeted preventive strategies for women of Asian ancestry.

### Supplementary Information

**Additional file 1: **Results for confounder selection analyses and sensitivity analysis using MLO view images.

## Data Availability

Datasets described and analysed in this manuscript are available from the corresponding author on reasonable request.
